# Effects of TRPC1 on epithelial mesenchymal transition in human airway in chronic obstructive pulmonary disease

**DOI:** 10.1097/MD.0000000000008166

**Published:** 2017-10-27

**Authors:** Feng Xu, Xiao-Chun Liu, Li Li, Chao-Nan Ma, Ya-Jun Zhang

**Affiliations:** a Department of Respiration, Huaihe Hospital of Henan University; b College of Nursing and Health, Henan University, Kaifeng, P.R. China.

**Keywords:** chronic obstructive pulmonary disease, cigarette smoking extract, E-cadherin, epithelial mesenchymal transition, human airway epithelial cells, pulmonary function, transient receptor potential canonical 1, vimentin

## Abstract

**Background:**

We investigated the effects of TRPC1 on epithelial mesenchymal transition (EMT) in human airway in chronic obstructive pulmonary disease (COPD).

**Methods:**

A total of 94 patients who underwent lobectomy were selected and divided into COPD (49 cases) and control (45 cases) groups. Immunohistochemistry was applied to detect expression of E-cadherin and vimentin and TRPC1. Correlation of TRPC1 expression with E-cadherin and vimentin expression, and correlations of lung function indicators in COPD patients with expression of TRPC1, E-cadherin, and vimentin were analyzed. Human airway epithelial cells (16HBE) were used for cell experiments; and cigarette smoking extract (CSE) was adopted to establish the COPD model using TRPC1 recombinant plasmids and siRNA. Cells were assigned into the control, CSE, CSE + vector, CSE + TRPC1, CSE + si-NC, and CSE + si-TRPC1 groups. Quantitative real-time polymerase chain reaction (qRT-PCR) and Western blot were implemented to detect expression of TRPC1, E-cadherin, and vimentin.

**Results:**

Compared with the control group, expression of TRPC1 and vimentin significantly increased while expression of E-cadherin decreased in the COPD group, and protein expression of TRPC1 was positively correlated with the protein expression of vimentin but negatively correlated with the protein expression of E-cadherin. Patients exhibiting positive expression of TRPC1 had lower FEV1, FEV1%Pred, and FEV1/FVC, compared with the patients exhibiting negative expression of TRPC1. Compared with the control group, expression of TRPC1 and vimentin increased, whereas expression of E-cadherin decreased in the CSE, CSE + vector, CSE + TRPC1, and CSE + si-NC groups. Compared with the CSE and CSE + vector groups, the expression of TRPC1 and vimentin increased but the expression of E-cadherin decreased in the CSE + TRPC1 group. Compared with the CSE and CSE + si-NC groups, the expression of TRPC1 and vimentin decreased but the expression of E-cadherin increased in the CSE + si-TRPC1 group. No significant differences were observed among the CSE, CSE + vector and CSE + si-NC groups.

**Conclusion:**

Overexpression of TRPC1 in COPD promoted EMT process and TRPC1 may be a new and interesting focus for COPD new treatment in the future.

## 1 Introduction

Chronic obstructive pulmonary disease (COPD) is a preventable and treatable disease characterized by limited sustainable airflow.^[1]^ Chronic progressive dyspnea, cough, and sputum are the main clinical manifestations of COPD.^[2]^ Current data confirmed that the prevalence rate in a global scale for COPD patients over 40 reached a rate of 9% to 10%,^[3]^ and the overall death cause for COPD is expected to rise to third place by 2030.^[4]^ Smoking is the main determinant for COPD and other risk factors include occupational exposure, air pollution, respiratory infections, genetic factors, host factors, and nutritional status.^[5]^ The pathological changes of COPD include mucus hypersecretion, ciliary dysfunction, small airway remodeling, lung tissue destruction, and emphysema.^[6]^ Auxiliary exercise therapy, drug therapy, and mechanical ventilation are the widely used therapeutic measures for COPD.^[7–9]^

Recent studies have demonstrated significant epithelial mesenchymal transition (EMT) in airway epithelial cells of COPD patients.^[10,11]^ EMT is the process in which polarized epithelial cells gain the characteristics of the interstitial cells through multiple biochemical changes. The formation process was represented by the decrease or disappearance of cell adhesion, loss of polarity and enhancement of cell motility, and migration.^[12]^ J Milara et al suggested in their study that the occurrence of EMT might be an important factor for the thickening and fibrosis of small airway wall in COPD patients.^[13]^ Furthermore, transient receptor potential (TRP) canonical proteins were considered to be important receptor cell membrane proteins and were involved in a variety of physiological body response processes. There are 7 TRPC subfamily isoforms (TRPC1-TRPC7) that could be activated by a variety of inflammatory cytokines and membrane stretch stimulus.^[14]^ It was reported that there was abundant TRPC1 protein expression in airway epithelial cells, showing an obvious increase of patients with chronic airway inflammatory disease compared with the normal patients.^[15]^ Thus, this study explored the impact of TRPC1 on EMT occurrence in airway epithelial cells of COPD patients with the least expectation to provide a theoretical basis for the study of COPD mechanism.

## 2 Materials and methods

### 2.1 Ethical statement

This study was approved by the clinical management committee of the Huaihe Hospital of Henan University. Informed written consents were obtained from all patients participating in this study. All procedures were conducted strictly in accordance with the Declaration of Helsinki involving human beings.

### 2.2 Study subjects

A total of 94 COPD patients who underwent lobectomy from January 2013 to June 2015 in Huaihe Hospital of Henan University were selected as study subjects. The study included 56 males and 38 females, from 42 to 77 years old with a calculated mean age of 59.1 ± 9.58. Preoperative pulmonary function test were implemented according to the spirometer measurement standard guidelines.^[16]^ To reach the maximum respiratory capacity, patients were seated with their noses clamped and instructed to breathe softly a few times before an end-expiratory inspiration. Then, the patients were instructed to expire forcefully to obtain the forced vital capacity (FCV) curve. The inspiration and expiration process was repeated 3 times to obtain the best FCV data showing differences <100 mL. The same technician operated this practical test with the same Jaeger spirometer to measure data. All patients were advised against the use of short-acting bronchodilators for a duration of 12 hours before the test (excluding patients suffering from asthma). Patients were divided into the COPD group (49 cases) and control group (45 cases) according to the test results referring to the 2011 diagnostic criteria of global initiative on COPD (GOLD).^[2]^ Lung tissue samples with a diameter of 1.5 cm (5 cm away from the lesions) were extracted and subsequently fixed in formalin for further use.

### 2.3 Embedded sections of lung tissues

The fixed lung tissues were respectively immersed in 50% ethanol for 5 hours, 75% ethanol for 5 hours, 85% ethanol for 2 hours, 95% ethanol I for 1 hour, 95% ethanol II for 1 hour, 100% ethanol I for 30 minutes and in 100% ethanol II for 30 minutes. It was followed by immersion in xylene I for 30 minutes, xylene II for 30 minutes, dipped wax I for 1 hour, dipped wax II for 1 hour, and dipped wax III for 1 hour. Tissues immersed in dipped wax were then sliced and placed into an embedded block that would be fixed on the wood block after cooling and be marked afterward. The marked paraffin blocks were sliced into 4-m thick sections (Leica, Germany). The spreading slices were dried at room temperature, baked under 65°C for 2 hours and then stored at room temperature for further use.

### 2.4 Immunohistochemical staining

Immunohistochemistry kit and diaminobezidin (DAB) color kits (ZSGB-Bio, China) were employed in the study and all operations were conducted according to instructions of the aforementioned kits. The paraffin slices were baked for 30 minutes at 60°C, and then placed in xylene and in different concentrations of ethanol to implement the dewaxing process. Ten minutes of incubation in 10% H_2_O_2_ at room temperature was carried out after tris-buffered saline (TBS), after TBST was immersed into citrate buffer solution, the mixture was heated until boiled by microwave. Thereafter the sliced tissues were preserved for 15 minutes at low temperature and then maintained around 92°C to 98°C, followed by cooling process at room temperature. The slices were incubated at 4°C overnight with the following primary antibodies after 20 minutes of goat serum closure: rabbit-anti-human TRPC1 polyclonal antibody (diluted at a ratio of 1:100, sc-20100, Santa Cruz Biotechnology, Santa Cruz, CA); rabbit-anti-human E-cadherin monoclonal antibody (diluted at a ratio of 1:500, ab40772, abcam Inc., Cambridge, MA); rabbit-anti-human vimentin monoclonal antibody (diluted at a ratio of 1:200, ab92547, abcam Inc., Cambridge, MA). Next day, the slices were rinsed in phosphate buffer saline (PBS) and incubated for 40 minutes in a humid box after drops of second antibody. Then the slices were washed by PBS and supplemented with horseradish peroxidase followed by incubation for 30 minutes. DAB coloration, ethanol dehydration, xylene transparence, and neutral resin sealing were carried out subsequently. The primary antibody was substituted by TBS to prepare the negative control group. Ten visual fields were randomly selected for the slices observed under OLYMPUS IX81 light microscope (Olympus, Tokyo, Japan) at a magnification of 200×. The dark brown cells were regarded as positive cells, and the percentage of cells were recorded as (−) for 0% to 10%, (+) for 10% to 25%, (++) for 25% to 50%, (+++) for 50% or more.^[17]^ (−) was regarded as negative expression, and (+), (++), and (+++) were regarded as positive expression. Image-Pro Plus 6.0 software (Media Cybernetics, Silver Spring, MD) was employed to detect staining intensity for further analysis.

### 2.5 Establishment of TRPC1 eukaryotic expressive vector and TRPC siRNA

After amplification by reverse transcription-polymerase chain reaction (RT-PCR), TRPC1 nucleic acid fragment at 455 bp was collected and purified. A reaction solution was added into the collected products using the pGM-T clone kit (Tiangen, Beijing, China), and the products were mixed with the vector at a ratio of 5:1. Subsequently, other components in the connection system were supplemented with the products, and the products were placed in the PCR appliance at 16°C for a night. After connected products were transformed into competent cell on the next day, the cells were inoculated in an LB agar culture medium (containing 5-bromo-4-chloro-3-indolyl-β-d-galactoside [xgal], isopropy-β-d-thiogalactoside [IPTG], and ampicillin) for culturing at 37°C overnight. The following day, the white bacterial colony was selected and placed in the LB culture medium containing ampicillin at 37°C, and then the mixture was shook for amplification. Plasmid DNA was extracted from the bacterial colony solution using a kit (Tiangen, Beijing, China) for transfection. The collected plasmid was carved by EcoR I incision at 37°C in a water bath, and the integrity of plasmid was analyzed using agarose gel electrophoresis. The empty plasmids were regarded as the control group. TRPC1 siRNA and NC siRNA were synthetized by Ribo Biotechnology Co, Ltd, Guangzhou, and the detailed sequence are shown as follows: TRPC1 siRNA: Sense strand: GGAUGUGCGGGAGGUGAAGTT; Antisense strand: CUUCACCUCCCGCACAUCCTT; NC siRNA: Sense strand: UUCUCCGAACGAACGUGUCACGUTT; Antisense strand: ACGUGACACGUUCGGAGAATT.

### 2.6 Preparation for cigarette smoking extract

Human bronchial epithelial 16HBE cells were obtained from the cell bank of the central laboratory of Huaihe Hospital of Henan University. The 16HBE cells cultured by DMEM culture medium (containing 10% fetal bovine serum [FBS]) were incubated at 37°C in a humidified incubator containing 5% CO_2_ in air. COPD models were established according to the cigarette smoking extract (CSE) method as follows: a cigarette burning without filter tip was aspirated continuously using a negative pressure suction device; smoke was drawn into 20 mL DMEM culture medium without serum to make the suspension. pH value was adjusted to 7.4 using 1 mol/L NaOH, and filtration and sterilizing were conducted using a 0.22 μm filter. The collected suspension was regarded as 100% CSE. CSE was used within 30 minutes; before using, CSE was diluted to 5% by culture medium without serum, and then 16HBE cells were treated by 5% CSE. Cell morphology was observed under an inverted microscope at 0-, 24-, 48-, 72-, and 96-hour time periods.

### 2.7 Cell grouping and transfection

The 16HBE cells in the logarithmic phase of growth were collected and inoculated in a 6-well plate (each well containing 2 × 10^5^ cells), and COPD models were established by CSE. The cells were treated by 5% CSE and subsequently transfected according to the instructions of the Lipofectamine 2000 reagent (Life Technologies, Gaithersburg, MD). The cells were inoculated in 6-well plates with each well containing 2 × 10^5^ cells and divided into the control group (16HBE cells without any treatment); CSE group (16HBE cells treated by 5% CSE for 48 hours); CSE + vector group (16HBE cells treated by 5% CSE for 48 hours followed by pGM-T vector transfection [Tiangen, Beijing, China]); CSE + TRPC1 group (16HBE cells treated by 5% CSE for 48 hours followed by pGM-T-TRPC1 recombinant plasmid transfection); CSE + si-NC group (16HBE cells treated by 5% CSE for 48 hours followed by NC siRNA transfection); CSE + si-TRPC1 group (16HBE cells treated by 5% CSE for 48 hours followed by TRPC1 siRNA transfection). The culture medium was replaced with 1640 culture medium containing 10% FBS for culturing after 6 hours. Further steps were taken after 48 hours.

### 2.8 Fluorescence quantitative reverse transcription-polymerase chain reaction

A TRIzol Kit (Invitrogen, Carlsbad, CA) was employed to extract cellular RNA from each group. An ultraviolet spectrophotometer (Eppendorf, Germany) was used to detect the purity and concentration of the test samples. RNA samples weighing 1000 ng would be extracted in accordance with the PrimeScript^TM^ RT Regeant Kit instructions (TAKARA, Japan) and mixed with 4 μL 5 × PrimeScriptTM Buffer, 1 μL PrimeScript^TM^ RT Enzyme Mix I, 1 μL oligo dT primer (50 μM), 1 μL random 6 mers (100 μM), and RNase free dH_2_O to construct a 20 μL reverse transcription system. The conditions were as follows: 30 minutes of reverse transcription at 37°C and 6 seconds of reverse transcription at 85°C. Reverse transcribed product of 1 μL was extracted and mixed with 4 μL 5 × Buffer, 0.6 μL dNTP (10 mM), 1.6 μL Primer Mix (10 μM), 0.8 μL Platinum TaqDNA Polymerase High Fedility, and 12 μL deionized water to construct a 10 μL PCR reaction system, whose reaction conditions were as follows: 2 minutes of PCR at 95°C, denaturation at 95°C for 45 seconds, annealing at 55°C for 45 seconds, and extension at 72°C for 2 minutes (30 cycles in total), followed by extension at 72°C for 10 minutes. Ten microliter PCR products were extracted and implemented with electrophoresis in 1.5% agarose gel supplemented with ethylene dibromide (EB). Images of PCR products were observed using an UV transmittance analyzer. Bandscan 5.0 was used to apply gray-scale analysis. The ratio of gray values of target genes and glyceraldehyde-3-phosphate dehydrogenase (GAPDH) was considered to be the relative intensity expression of genes. The upstream and downstream primer sequences for each gene are shown in Table 1.

**Table 1:**
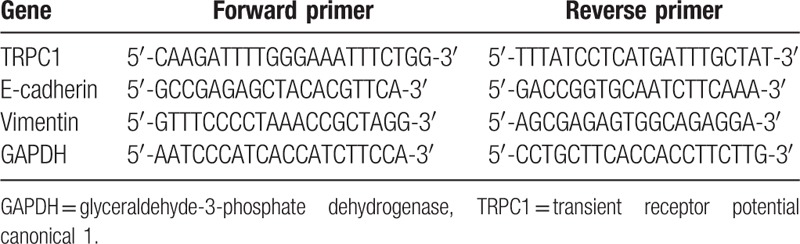
Primer sequences.

### 2.9 Western blot assay

Total cellular protein in each group was extracted in accordance with the specifications of the RIPA cell lysate (ShineGene Molecular Biotechnology, China) and implemented for protein quantification using a BCA Protein Assay Kit (Beyotime Biotechnology, China). SDS-PAGE rubber was prepared with the calculation of protein concentration according to the sample volume, and mixture of sample buffer at a ratio of 1:1 was conducted afterward with 5 minutes of boiling water bath and addition of extra samples. Electrophoresis was implemented respectively with 5% stacking gel, 12% separation gel at voltages of 80 V and 100 V. The gels was marked with cutting corners and summated with 2 hours of electrophoretic transference under 200 mA of constant current electroporation condition in a cold room at 4°C. TBST membrane was blocked in 5% skim milk and then washed 3 times after 1 hour of preservation at room temperature, 10 minutes each time, followed by immersion in TRPC1 rabbit anti-human monoclonal antibody at a dilution ratio of 1:10000 (ab51255, abcam Inc., Cambridge, MA); rat anti-human E-cadherin monoclonal antibody at a dilution ratio of 1:50 (ab1416, abcam Inc., Cambridge, MA); mouse anti-human vimentin monoclonal antibody at a dilution ratio of 1:500 (ab8978, abcam Inc., Cambridge, MA) and rabbit anti-human GAPDH polyclonal antibody at a dilution ratio of 1:4000 (ab129348, abcam Inc., Cambridge, MA) to incubate at 4°C overnight. The TBST membrane was washed 3 times the following day, 10 minutes each time. Incubation would be implemented for 1 hour in the HRP-labeled IgG (abcam Inc., Cambridge, MA) at room temperature. ImageJ software would be applied to analyze the obtained strip data after gel imaging system scanning process and ECL kit colorization after membrane washing.

### 2.10 Statistical analysis

Statistical analyses were performed using the SPSS21.0 software (SPSS Inc, Chicago, IL). Measurement data were represented as mean ± standard deviation (SD). Unpaired *t* test was used for comparisons between 2 groups and one-way analysis of variance (ANOVA) was used for comparisons among multiple. Numeration data were represented as number, percentage, or rate. The Spearman rank correlation test was used to detect the expression correlation of each protein, and the grade rank sum test was applied to compare the immunohistochemistry results of each group. Comparisons of effects of CSE on EMT markers in 16HBE cells were conducted by repeated measurement ANOVA. All tests were 2-sided and *P* < .05 was considered statistically significant.

## 3 Results

### 3.1 Comparison of baseline characteristics

The comparison results of patients’ basic characteristics and lung function were shown in Table 2. Comparisons between COPD group and control group show that there were significant differences in smoking volume, FEV1, FEV1%Pred, FEV1/FVC (all *P* < .05), and there were no obvious differences in sex, age, type of disease, smoking volume, body mass index, and FVC (all *P* > .05).

**Table 2:**
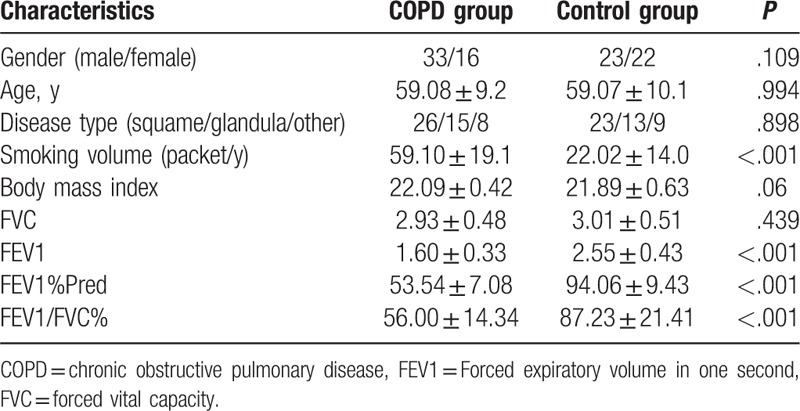
Baseline characteristics of patients in the COPD and control groups.

### 3.2 Correlation analysis on the expressions of TRPC1 with vimentin and E-cadherin

**Figure 1:**
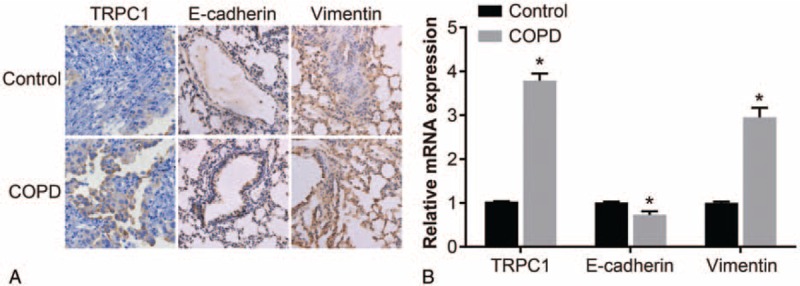
Correlation as well as mRNA and protein expression of TRPC1, vimentin, and E-cadherin. Note: (A) immunohistochemistry images of expression of TRPC1, vimentin and E-cadherin (×400); (B) mRNA expression of TRPC1, vimentin, and E-cadherin detected using RT-PCR. COPD = chronic obstructive pulmonary disease, EMT = epithelial mesenchymal transition, RT-PCR = reverse transcription-polymerase chain reaction, TRPC1 = transient receptor potential canonical 1.

Results of immunohistochemistry and quantitative real-time polymerase chain reaction (qRT-PCR) show that positive cells were the immuno-reactive products presented by brown-yellow in nucleus and cytoplasm (Fig. 1A). Statistical analysis indicated that compared with the control group, mRNA and protein expression of TRPC1 and vimentin significantly increased whereas mRNA and protein expression of E-cadherin decreased (*P* < .05) (Fig. 1B, Table 3). Results of Spearman rank correlation test demonstrate that expression of TRPC1 was positively correlated with vimentin protein expression (*r* = 0.265, *P* < .05), and negatively correlated with E-cadherin protein expression (*r* = −0.206, *P* < .05) (Table 4).

**Table 3:**
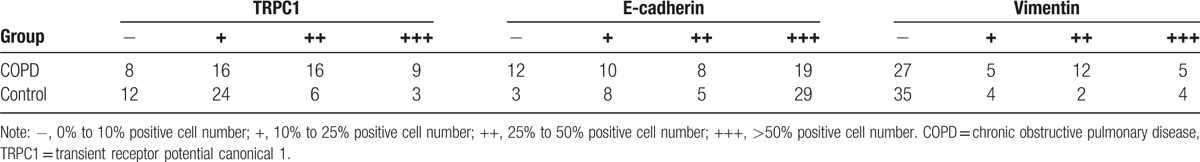
Expressions of TRPC1, vimentin, and E-cadherin detected by immunohistochemistry.

**Table 4:**
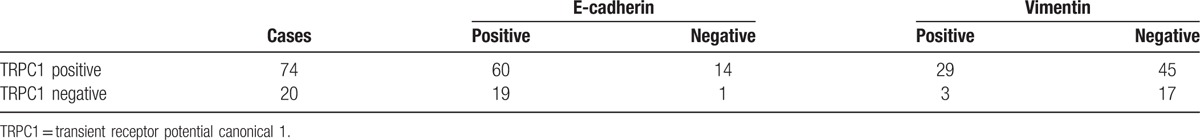
E-cadherin and vimentin expressions of positive and negative TRPC1 patients.

### 3.3 Correlation between the expressions of TRPC1, vimentin, and E-cadherin and Lung function index

All included patients were assigned into the positive and negative group based on the protein expression of TRPC1, and the results are shown in Table 5. There were no significant differences in FVC values among the positive and negative groups (*P* > .05), whereas values of FEV1, FEV1%Pred, and FEV1/FVC were lower compared with the negative group (*P* < .05).

**Table 5:**
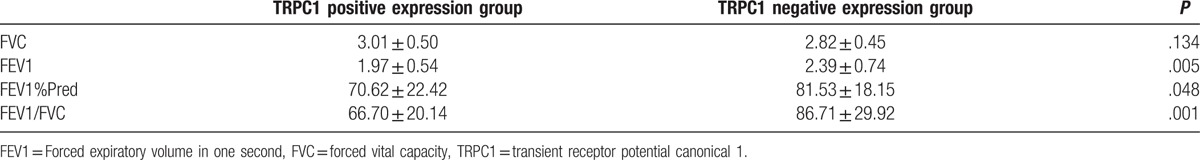
Lung function index comparison of different TRPC1 patients.

### 3.4 Effect of CSE on airway epithelium morphology and EMT markers

**Figure 2:**
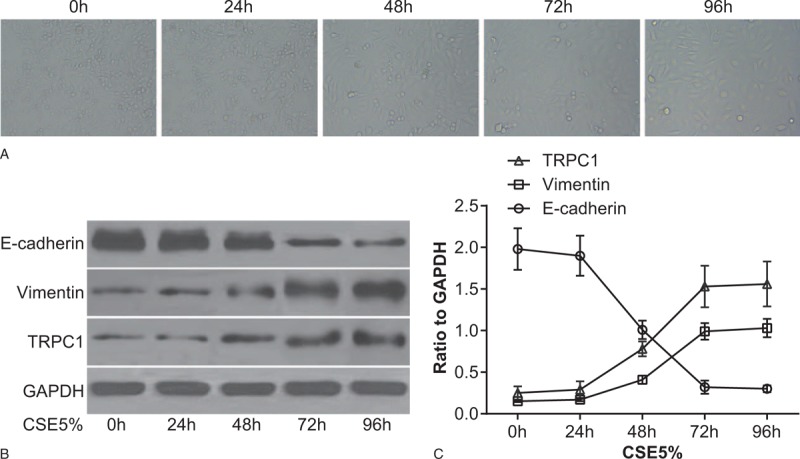
Effect of CSE on 16HBE cell morphology, E-cadherin and vimentin. Note: (A) effect of CSE on 16HBE cell morphology observed under an inverted microscope at different time periods (observed at a magnification of 200×); (B) effect of CSE on E-cadherin and vimentin in 16HBE cells detected by Western blot assay at different time periods, compared with the 0 h, *P* < .05, compared with the 24 h time period, *P* < 0.05, compared with the 48 h time period, *P* < .05. CSE = cigarette smoking extract, GAPDH = glyceraldehyde-3-phosphate dehydrogenase, TRPC1 = transient receptor potential canonical 1.

After 16HBE cells were treated by 5% CSE, changes in cell morphology were observed using an inverted microscope, and findings indicate that 16HBE cells attained a cuboidal or pebble shape, characterized by typical cobblestone features and cells were closely connected at the 0- and the 24-hour time periods. At the 48-hour time periods, cells were observed to be enlarged with wider cell gaps and elongated into a fusiform shape, and cells were loosely connected, and furthermore, no significant necrosis and apoptosis were observed in the 16HBE cells until the 96-hour time periods (Fig. 2A). Results of Western blot found protein expression of TRPC1, E-cadherin, and vimentin in 16HBE cells after being treated with 5% CSE; there were no significant differences on expression of TRPC1, E-cadherin, and vimentin at the 0 and 24 hours (*P* > .05). Compared with the 0- and 24-hour time periods, the protein expression of TRPC1 and vimentin increased whereas protein expression of E-cadherin decreased at the 48-, 72-, and 96-hour time periods; compared with the 48 hours, at the 72 hours, the protein expression of TRPC1 and vimentin increased whereas protein expression of E-cadherin decreased (both *P* < .05). No significant differences in the protein expression of TRPC1, E-cadherin, and vimentin were observed among the 72- and 96-hour time periods (*P* > .05) (Fig. 2B). Based on the aforementioned results, 16HBE cells treated with 5% CSE for 48 hours were selected for subsequent cell transfection experiments.

### 3.5 Cell morphology and expression of TRPC1 after transfection

**Figure 3:**
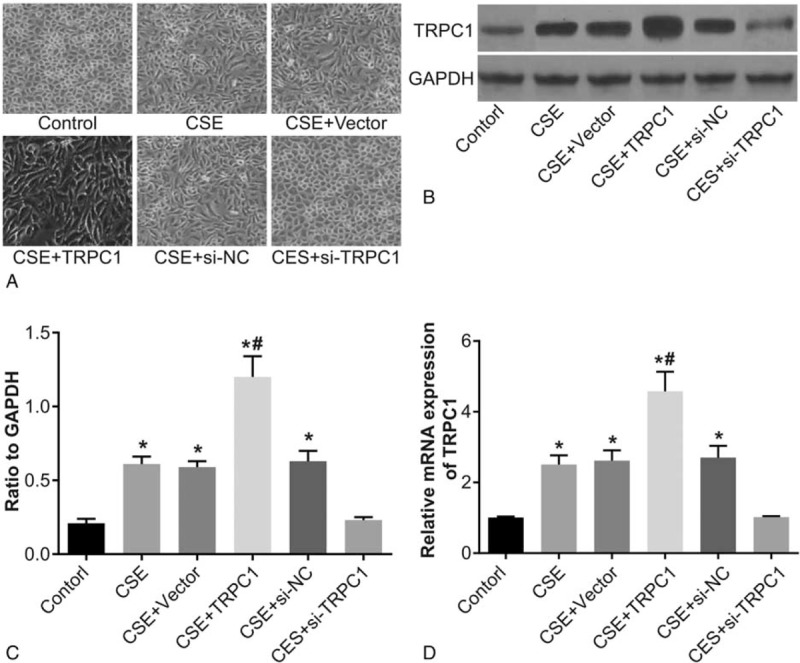
Cell morphology after transfection and expression of TRPC1. Note: (A) cell morphology after transfection observed under an inverted microscope (observed at a magnification of 200×); (B) protein expression in cells determined by Western blot assay; (C) histogram of protein expression levels for each group after transfection; (D) histogram of mRNA expression levels for each group after transfection. ∗, compared with the control group, *P* < .05; ^#^, compared with the CSE, CSE + vector, and CSE + si-NC groups, *P* < .05. CSE = cigarette smoking extract, GAPDH = glyceraldehyde-3-phosphate dehydrogenase, TRPC1 = transient receptor potential canonical 1.

Morphology of 16HBE cells were observed under an inverted microscope and it was found that after transfection, monolayer cells of the control group and the CSE + si-TRPC1 group transformed into cuboidal or pebble-shaped cells, characterized by typical cobblestone features, and cells were closely connected. Some cells in the CSE, CSE + vector, and CSE + si-NC groups were observed to be enlarged with wider cell gaps and the cells elongated into a fusiform shape, losing their original paving stones growth pattern, and significant changes were observed in the CSE + TRPC1 group (Fig. 3A). Results of Western blot assay and RT-PCR show that compared with the control group, protein and mRNA expression of TRPC1 significantly increased in the CSE, CSE + vector, CSE + TRPC1 and CSE + si-NC groups (both *P* < .05). No significant differences in the mRNA and protein expression of TRPC1 were observed among the CSE + si-TRPC1 and control groups. Compared with the CSE and CSE + vector groups, mRNA and protein expression of TRPC1 increased in the CSE + TRPC1 group, and there were no significant difference in the mRNA and protein expression of TRPC1 among the CSE, CSE + vector, and CSE + si-NC groups (both *P* > .05) (Fig. 3B and D).

### 3.6 Expression of vimentin and E-cadherin after TRPC1 transfection

**Figure 4:**
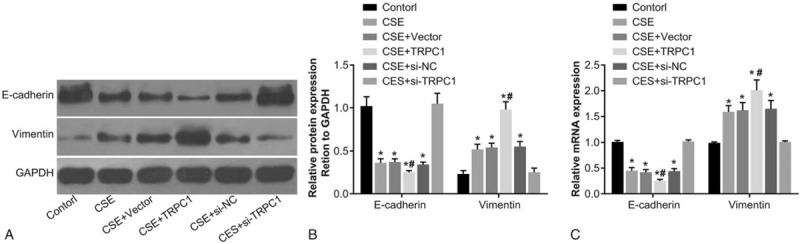
Protein expression of E-cadherin and vimentin in each group detected by Western blot assay and RT-PCR posttransfection. Note: (A) protein expression of E-cadherin and vimentin after transfection; (B) histogram of protein expression of E-cadherin and vimentin after transfection; (C) histogram of mRNA expression of E-cadherin and vimentin determined by RT-PCR after transfection. ^∗^, compared with the control group, *P* < .05; ^#^, compared with the CSE + vector and CSE + si-NC groups, *P* < .05. CSE = cigarette smoking extract, GAPDH = glyceraldehyde-3-phosphate dehydrogenase, RT-PCR = reverse transcription-polymerase chain reaction, TRPC1 = transient receptor potential canonical 1.

Results of Western blot assay and RT-PCR showed that compared with the control group, the mRNA and protein expression of E-cadherin decreased in the CES, CSE + vector, CSE + TRPC1, and CSE + si-NC groups, whereas expression of vimentin was found to be increased (*P* < .05). No significant differences in the expression of E-cadherin and vimentin were observed among the CES + si-TRPC1 and control group (*P* > .05). Compared with the CES and CSE + vector groups, the protein and mRNA expression of E-cadherin decreased in the CSE + TRPC1 group, whereas the protein and mRNA expression of vimentin was found to be increased. Compared with the CSE and CSE + si-NC group, the protein and mRNA expression of E-cadherin increased in the CSE + si-TRPC1 group, whereas the protein and mRNA expression of vimentin was found to be decreased (*P* < .05). There were no remarkable difference on mRNA and protein expressions of E-cadherin and vimentin among the CSE, CSE + vector, and CSE + si-NC groups (*P* > .05) (Fig. 4).

## 4 Discussion

Nowadays, the EMT mechanism for human airway remodeling in COPD has attracted the attention of various researchers. It has been reported that TGF-β1 plays an important role in COPD development through the Smad2/3 mechanism to activate EMT.^[18]^ Another report also indicated that upregulation of uPAR in small airway epithelial cells of COPD patients might be involved in the occurrence of EMT.^[19]^ In addition, TRPC1, a pressure receptor of airway epithelial cells, has reportedly been involved in the occurrence of COPD.^[20]^ However, the association mechanism of TRPC1 with the occurrence of EMT for COPD patients remains unclear and light needs to be shed on this mechanism for better treatment and prognosis of COPD patients. Therefore, this study explored the impact of TRPC1 on the occurrence of EMT for COPD airway epithelial cells.

The results of this study demonstrate that the TRPC1 and vimentin expression levels of lung tissue for COPD patients were higher compared with the control group, while the E-cadherin expression level was lower than the control group. The possible explanation might be that TRPC1 serves as an important mechanical sensitive ion channel in cells, which was further confirmed by J Berrout et al whose studies claim that TRPC1 was a pressure-sensitive ion channel in vascular endothelial cells.^[21,22]^ Pathological changes of airway remodeling for COPD patients might result in airflow limitation and high pressure in human airway.^[23]^ Under this circumstance, airway epithelial cells were stimulated to secrete large amounts of TGF-β1.^[23]^ Meanwhile, TGF-β1 was reported to upregulate the expression of TRPC1, whereas decrease the expression of E-cadherin mRNA.^[24,25]^ Gohy et al found that E-cadherin decreased in COPD airway.^[26]^ E-cadherin, a calcium-dependent epithelial adhesion molecule is considered to play an important role in maintenance of normal epithelial cell morphology and structural integrity.^[27]^ The induction or deficiency of E-cadherin expression could lead to the decrease of mutual adhesion of cells, which may be of significant importance in promoting the development of EMT.^[28]^ In addition, vimentin is a type of intermediate microfilament protein and is capable of changing cell shapes from cube to spindle when substituted as the main component of keratin cytoskeleton.^[29]^ Cytoskeleton transition was proved to be vimentin-based in the EMT process.^[30]^ Chen et al also demonstrated that upregulation of vimentin was observed in small airway epithelium of COPD lungs.^[31]^ Therefore, the occurrence of EMT was accompanied by an increased expression of vimentin. Jean's research demonstrated the increase of EMT was accompanied by the gradual disappearance of E-cadherin and elevated expression of vimentin.^[32]^ This result was consistent with the results of our study, indicating that EMT phenomenon was observed in COPD patients, leading to cell membrane wall fibrosis and thickening of the cell wall, as well as the prompt remodeling of COPD airway or persistent and progressive airflow limitation, so as to participate in the COPD development.^[33]^

The Spearman analysis indicated that the expression of TRPC1 was negatively correlated with E-cadherin expression and positively correlated with the vimentin expression of lung tissues for COPD patients. All of these indicate that the increase of TRPC1 was closely related to the occurrence of EMT in COPD patients. Madsen et al found that TRPC1 channel proteins involved in flow regulation of operative Ca^2+^, causing influx increase of extracellular Ca^2+^.^[34]^ Ca^2+^ is a key signaling molecule activated by multiple receptors in signal transduction process and could be involved in cell regulation. Ca^2+^ influx plays an important role in the process of EMT development.^[35]^ A further study confirmed that TRPC1 could induce the occurrence of EMT in 16HBE of airway epithelial cells by the addition of TRPC1 expression and upregulation of intracellular Ca^2+^ concentration. Ca^2+^ is capable of activating phospholipase C that could accelerate the decomposition of phosphatidylinositol 4,5-diphosphate into inositol triphosphate and diacyl glycerol.^[36]^

In summary, this study confirmed that TRPC1 is related to the occurrence of COPD and lung function index. In addition, TRPC1 expression levels also affect the expression levels of EMT protein markers. However, there were 2 limitations in the present study. First, TRPC1 silencing could inhibit the EMT occurrence of human airway epithelial cells 16HBE according to the results of this study. Nevertheless, there were no TRPC1 silencing group, thus we were unable to provide evidence to support this targeted therapy. Second, EMT development mechanism of TRPC1 requires further analyses and discussion, such as the specific inflammation factors involved in the occurrence of EMT for COPD patients. Therefore, further studies, analyses, and clinical validation of TRPC1 should be conducted for TRPC1 to be a new therapeutic target for COPD patients.

## Acknowledgments

We would like to acknowledge the helpful comments on this paper received from our reviewers.
